# ^1^H NMR serum metabolomic profiling of patients at risk of cardiovascular diseases performing stress test

**DOI:** 10.1038/s41598-020-74880-6

**Published:** 2020-10-20

**Authors:** Camila Lema, Mireia Andrés, Santiago Aguadé-Bruix, Marta Consegal, Antonio Rodriguez-Sinovas, Begoña Benito, Ignacio Ferreira-Gonzalez, Ignasi Barba

**Affiliations:** 1 Cardiovascular Diseases Research Group, Department of Cardiology, Vall d’Hebron University Hospital and Research Institute, Universitat Autònoma de Barcelona, Pg Vall d’Hebron 119-129, 08035 Barcelona, Spain; 2 Centro de Investigación Biomédica en Red Sobre Enfermedades Cardiovasculares (CIBER-CV), Madrid, Spain; 3 Centro de Investigación Biomédica en Red Sobre Epidemiología Y Salud Pública (CIBERESP), Madrid, Spain; 4 Vall D’Hebron Insitutute of Oncology (VHIO), Barcelona, Spain

**Keywords:** Acute coronary syndromes, Metabolomics

## Abstract

Cardiovascular diseases are the leading cause of death worldwide. Changes in lifestyle and/or pharmacological treatment are able to reduce the burden of coronary artery diseases (CAD) and early diagnosis is crucial for the timely and optimal management of the disease. Stress testing is a good method to measure the burden of CAD but it is time consuming and pharmacological testing may not fully mimic exercise test. The objectives of the present project were to characterize the metabolic profile of the population undergoing pharmacological and exercise stress testing to evaluate possible differences between them, and to assess the capacity of ^1^H NMR spectroscopy to predict positive stress testing. Pattern recognition was applied to ^1^H NMR spectra from serum of patients undergoing stress test and metabolites were quantified. The effects of the stress test, confounding variables and the ability to predict ischemia were evaluated using OPLS-DA. There was an increase in lactate and alanine concentrations in post-test samples in patients undergoing exercise test, but not in those submitted to pharmacological testing. However, when considering only pharmacological patients, those with a positive test result, showed increased serum lactate, that was masked by the much larger amount of lactate associated to exercise testing. In conclusion, we have established that pharmacological stress test does not reproduce the dynamic changes observed in exercise stress. Although there is promising evidence suggesting that ^1^H NMR based metabolomics could predict stress test results, further studies with much larger populations will be required in order to obtain a definitive answer.

## Introduction

Cardiovascular diseases are the leading cause of death worldwide^1^, being coronary artery disease (CAD) one of the most prevalent cardiovascular disorders. Changes in lifestyle and/or pharmacological treatment are able to reduce the burden of CAD, so early diagnosis becomes crucial for a timely and optimal management of the disease^2^. At present, the risk of CAD can be evaluated by the presence of established risk factors (including age, sex, hypertension, hypercholesterolemia, obesity, diabetes mellitus, sedentary lifestyle and smoking) in tables such as the Framingham Risk Score and the Adult Treatment Panel III, SCORE (Systematic Coronary Risk Evaluation)^3,4^. In patients at risk of CAD, confirmation of the disease is established by invasive coronary angiography, and more recently, by angio-coronary computed tomography (CT) scan, showing obstructive plaques at the coronary lumen. These two techniques, considered the gold-standard diagnostic tools for CAD, are however limited by their risks (particularly those inherent to an invasive procedure in the case of coronary angiogram), radiation exposure, and costs. Other cardiac noninvasive techniques such as stress echocardiography, cardiac magnetic resonance imaging (MRI), and single-photon emission computed tomography (SPECT)^5,6^ have been used to predict (with moderate ability) the presence of CAD. While nuclear stress testing has sensitivity above 80% and specificity that reaches 95%^7^, it is also time consuming and not adequate for population screening. Furthermore, it is not clear if pharmacological stress applied to the patients that cannot exercise has the same clinical interpretation as exercise testing.

There is considerable interest in developing personalized, non-invasive methods for cardiovascular risk stratification that could be applied at the population level. Metabolomics has the potential to play a leading role in the development of personalized medicine^8,9^. In recent years there has been an effort to develop novel strategies based on ^1^H NMR profiling of serum for the personalized diagnosis and prognosis of CAD^10^. However, the results have been of limited value^11^ and the approach has not yet reached clinical practice^12^.

Low-molecular-weight metabolites are relevant to exercise physiology and myocardial ischemia, and therefore studies with different approaches have been carried out to develop or improve current tools for risk stratification of patients with suspected CAD. Sabatine et al. showed that metabolites such as lactate and alanine, and particularly compounds related to the citric acid pathway, are increased in the setting of myocardial ischemia^13^. Using a ^1^H NMR-based approach in patients undergoing stress test, our group described that the main contributors to discriminate between ischemia (post-exercise) and baseline (pre-exercise) samples were lactate, glucose, methyl and methylene moieties of lipids and long-chain amino acids (valine, leucine, and isoleucine)^14^. More recently, a study conducted in the emergency department showed that acylcarnitines were associated with increased acute coronary syndrome risk^15^.

The objectives of the present project were two fold; first, we evaluated the metabolic profile and dynamics over time of patients undergoing a pharmacological stress test compared to those undergoing exercise stress test on a treadmill; secondly, we evaluated the capacity of ^1^H NMR spectroscopy to predict positive testing in both modalities of stress (pharmacological and exercise).

## Results

### Epidemiological and clinical characteristics of the patients

Of 244 consecutive patients referred for exercise or pharmacological test, 32 were excluded because of insufficient exercise and need to complete the test with pharmacological stress (mixed stress test). Of the 212 eligible patients, 83 underwent a pharmacological test and 86 underwent an exercise test. Out of the patients undergoing pharmacological test, 20 had a positive result (cases), and 23 age- and sex-matched controls (with negative result) were selected. Among patients undergoing exercise test, 39 were positive (cases) and 44 age- and sex-matched controls were selected.

Table 1 summarizes the epidemiological and clinical characteristics of the overall population, as well as the results of the stress tests. Most patients were men (76.2%), and the mean age was 67.2 ± 10.7 years. The prevalence of cardiovascular risk factors was high, with more than 65% of patients being current or former smokers, almost 70% having hypertension, more than 80% having dyslipidemia and one third being diabetic. Up to 50% of patients had a history of previous CAD, in 82% of cases with previous coronary revascularization.

**Table 1:** Epidemiologic and demographic characteristics of the patients included in the study.

Characteristics	Total	Exercise stress test	Pharmacological stress test	*P*
	(N = 126)	(N = 83)	(N = 43)	
**Demographics**				
Sex, men	96 (76.2%)	73 (88.0%)	23 (53.5%)	< 0.01
Age (years)*	67.2 ± 10.7	63.8 ± 10.2	73.7 ± 8.8	< 0.01
**Cardiovascular risk factors**				
Smoking	(n = 110)	(n = 73)	(n = 37)	0.09
Ex smoker	62 (56.4%)	46 (63.0%)	16 (43.2%)	
Smoker	12 (10.9%)	8 (11.0%)	4 (10.8%)	
Non smoker	36 (32.7%)	19 (26.0%)	17 (45.9%)	
Hypertension	87 (69.0%)	52 (62.7%)	35 (81.4%)	0.06
Dyslipidemia	106 (84.1%)	72 (86.7%)	34 (79.1%)	0.20
Diabetes mellitus	42 (33.3%)	23 (27.7%)	19 (44.2%)	0.08
Non insulin requirement	26 (61.9%)	16 (19.3%)	10 (23.3%)	
Insulin requirement	16 (38.1%)	7 (8.4%)	9 (20.9%)	
**Prior cardiovascular disease**				
CAD	63 (50.0%)	45 (54.2%)	18 (41.9%)	0.26
Coronary revascularization	52 (82.5%)	40 (88.8%)	12 (66.7%)	0.12
Cerebrovascular disease	7 (5.6%)	3 (3.6%)	4 (9.3%)	0.23
Chronic kidney disease	23 (18.2%)	6 (7.2%)	17 (39.5%)	< 0.01
Pneumopathy	22 (17.5%)	12 (14.4%)	10 (23.3%)	0.34
**Long-term medication**				
Statins	96 (76.2%)	65 (78.3%)	31 (72.1%)	0.37
Fibrates	5 (4.0%)	3 (3.6%)	2 (4.7%)	0.77
Ezetimib	9 (7.1%)	4 (4.8%)	5 (11.6%)	0.16
ASA	88 (69.8%)	64 (77.1%)	24 (55.8%)	0.01
Beta-blockers	72 (57.1%)	46 (55.4%)	26 (60.5%)	0.71
Nitrates	27 (21.4%)	16 (19.3%)	11 (25.6%)	0.41
Oral antidiabetics	34 (27.0%)	21 (25.3%)	13 (30.2%)	0.55
ACE inhibitor/ARB	71 (56.3%)	43 (51.8%)	28 (65.1%)	0.15
Antropometric and laboratory parameters*				
BMI (Kg/m^2)^	28.9 ± 4.6	29.2 ± 3.8	30.3 ± 5.6	0.02
Total cholesterol (mg/dl)	175.6 ± 43.1	172.7 ± 40.7	181.0 ± 47.2	0.31
LDL (mg/dl)	101.1 ± 36.0	101.6 ± 36.6	100.1 ± 35.6	0.21
HDL (mg/dl)	47.5 ± 12.3	46.6 ± 11.2	49.3 ± 13.5	0.26
Triglycerides (mg/dl)	130.9 ± 53.5	121.1 ± 42.1	149.7 ± 67.1	0.01
Glomerular filtration (mL/min/1.73 m^2^)	74.6 ± 20.3	80.1 ± 15.2	63.4 ± 24.6	< 0.01
**SPECT data**				
Indication				0.84
Diagnostic	57 (45.2%)	37 (44.6%)	20 (46.5%)	
Pronostic	69 (54.8%)	46 (55.4%)	23 (53.5%)	
METS	8.1 ± 2.0	8.1 ± 2.0	–	
Initial BP	138.1 ± 21.2	135.1 ± 18.6	143.8 ± 27.7	0.05
Maximum intensity BP	157.9 ± 31.7	167.5 ± 31.9	139.2 ± 21.6	< 0.01
Basal HR	71.2 ± 15.3	71.3 ± 15.8	71.1 ± 14.4	0.95
Maximal HR	114.8 ± 26.5	127.8 ± 20.5	89.8 ± 14.4	< 0.01
% Increase HR	65.9 ± 44.9	85.4 ± 41.6	28.1 ± 20.1	< 0.01
FE	55.9 ± 11.8	55.0 ± 11.2	57.5 ± 12.9	0.28
Positive test for Ischemia	59 (46.8%)	39 (47.0%)	20 (46.5%)	0.95

*Mean ± SD.

Table 1 also presents the characteristics of patients according to the two stress protocols. Women were more prevalent in the pharmacological group (46.5% vs 12.0% in the exercise stress group; *P* < 0.001). As expected, patients who performed a pharmacological stress test, compared to those who underwent an exercise test, were significantly older (73.7 ± 8.8 vs. 63.8 ± 10.2 years; *P* < 0.001), and had more comorbidities such as chronic kidney disease, higher body mass index (BMI) and a trend to higher prevalence of hypertension and diabetes.

### ^1^H NMR Spectrum characterization

**Figure 1:**
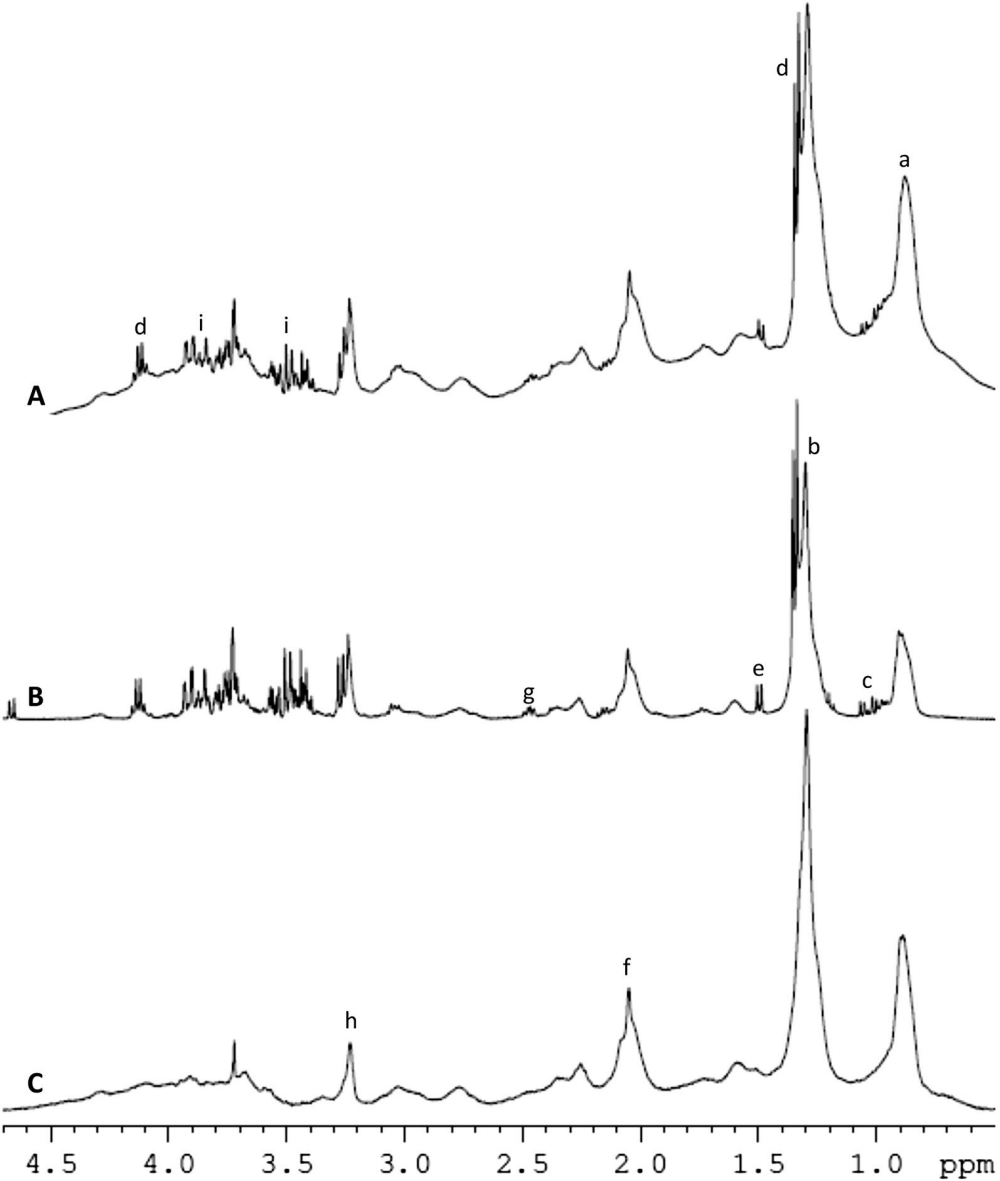
Typical serum spectra obtained from one patient using different pulse sequences. (**A**) pulse-and-acquire, (**B**) CPMG with an effective T2 delay of 32 ms and (**C**) diffusion edited spectra. Only the aliphatic part of the spectra is shown, spectra were acquired as described in the methods section. Tentative assignations based on chemical shift are as follows: (a) and (b) methyl end methylene groups of fatty acid chains in lipoproteins respectively; (c) Valine, leucine and isoleucine; (d) lactate; (e) alanine; (f) protons next to double bonds in fatty acid chains; (g) glutamate; (h) timethylamine containing compounds and (i) glucose.

Figure 1 shows the typical spectra of a whole serum sample obtained with different pulse sequences. The use of different pulse sequences allows to investigate different properties of the samples; spectra obtained with pulse-and-acquire (Fig. 1A) is dominated by large broad peaks corresponding to the methyl groups of lipids in lipoprotein centered at 0.9 ppm; a large peak of methylene protons at 1.28 ppm; a doublet corresponding to lactate at 1.35 ppm; and various peaks of glucose in the area between 3.2 and 3.9 ppm. The CPMG sequence (Fig. 1B) is T2-edited and removes the signal from large, slow tumbling, macromolecules. Thus, low molecular weight metabolites such as lactate, glucose and aminoacids at around 1 ppm are easier to detect. Finally, signals from low molecular weight metabolites are removed from the diffusion-edited spectra (Fig. 1C) and only the signals arising from the lipoprotein envelope remain.

### The effect of confounding variables

Visual inspection of the spectra did not allow identifying any major characteristics associated with the potential confounding factors including age, sex, dyslipidemia, DM or pharmacological treatment, particularly the use of statins. PCA, an unsupervised pattern recognition approach, did not show any clustering associated with the confounding variables.

**Figure 2:**
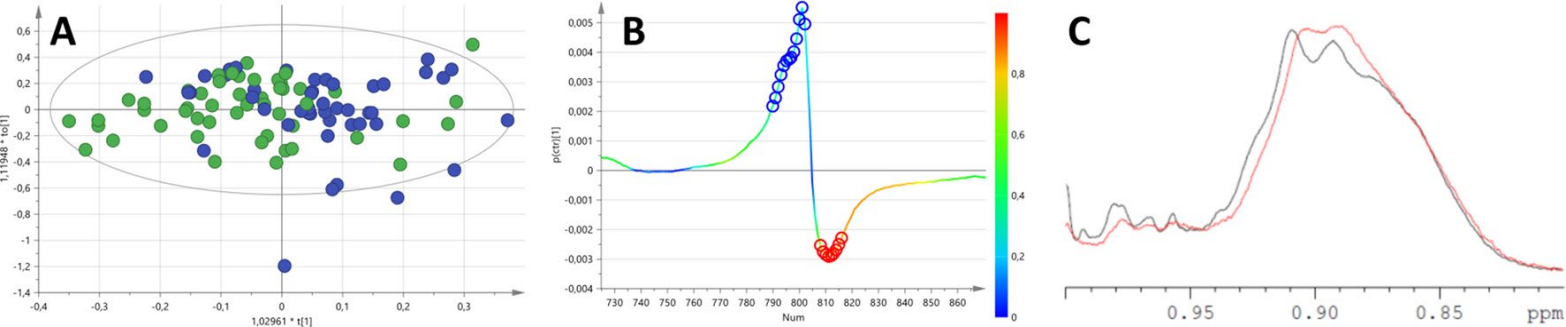
OPLS-DA models differentiating between men and women (**A**). The s-plot (**B**) shows that women lipid peaks tend to shift to high fields (blue circles), as also shown in the example of the methyl lipid peaks (**C**; red represent men, black represent women).

When applying supervised discriminant analysis (OPLS-DA), it was possible to discriminate between men and women using the spectra acquired with different pulse sequences (Characteristics of the different discriminant models are detailed in Table 2). Classification success was between 92 and 96% depending on the spectra. The main differences were found around the lipid area (1.28 and 0.9 ppm). Figure 2 corresponds to the OPLS-DA model (A) discriminating men from women. The most relevant variables of discrimination were found in the lipid region (Fig. 2B,C), and showed that it was the shape of the peaks, rather than their intensity, that was associated with sex discrimination, as women lipid peaks tended to shift to lower field (higher ppm values). Table 2 shows the characteristics of the discriminant models to differentiate between men and women obtained with different pulse-sequence spectra. Although deproteinized spectra were also able to differentiate between men and women (Table 2, NOESYPR1D), metabolite quantification obtained from the same spectra (Table 3) showed no differences in metabolite quantification once corrected for multiple comparisons.

**Table 2:** Characteristics of the OPLS-DA models obtained to differentiate between sexes.

Sequence	R^2^X	R^2^Y	Q^2^	Fisher	CV ANOVA
WG	0.77	0.49	0.30	1.4^−33^	1.5^−15^
CPMG	0.67	0.58	0.44	1.10^−38^	1.13^−26^
Diffusion	0.60	0.31	0.25	2.0^−13^	2.16^−14^
NOESYPR1D	0.55	0.15	0.09	1.0^−5^	4.04^−5^

**Table 3:** Metabolite concentration (mmol/L) derived from deproteinized spectra samples according to the sex.

Metabolite	Male	Female	*P*
3-Hydroxybutyrate	0.042 ± 0.025	0.044 ± 0.026	0.796
Acetate	0.037 ± 0.191	0.041 ± 0.083	0.953
Alanine	0.010 ± 0.053	0.009 ± 0.005	0.965
Betaine	0.025 ± 0.026	0.022 ± 0.015	0.408
Creatine	0.013 ± 0.010	0.018 ± 0.011	0.072
Creatinine	0.034 ± 0.028	0.027 ± 0.014	0.106
Glucose	1.793 ± 1.166	2.005 ± 0.095	0.324
Glycine	0.016 ± 0.022	0.023 ± 0.021	0.106
Isoleucine	0.064 ± 0.036	0.037 ± 0.004	0.106
Lactate	1.238 ± 0.802	1.123 ± 0.545	0.408
Threonine	0.147 ± 0.091	0.175 ± 0.101	0.106
Valine	0.102 ± 0.060	0.116 ± 0.068	0.216

*P* values were corrected for multiple comparisons.

Patients with diabetes mellitus could not be differentiated from controls by discriminant analysis in any of the spectra. However, it is worth noting that most diabetic patients were taking oral antidiabetic agents and no one showed a glucose level above 6 mmol/L at the time of analysis. Similarly, patients with dyslipidemia (most of them treated with statins) could not be discriminated. However, statin treatment induced a reduction in the height of the methylene lipid peaks –(CH_2_)_n_ at around 1.28 ppm and at around 2.0 ppm, the area were lipid double bonds appear, that could be detected in pulse-and-acquire and diffusion-edited spectra (Supplementary Fig. 1). However, the effect was lost in deproteinized and T2-edited spectra. Finally, we could not detect differences in the concentrations of low molecular weight metabolites associated with dyslipidemia or statin treatment (Supplementary Table 2).

### The effect of stress test

**Figure 3:**
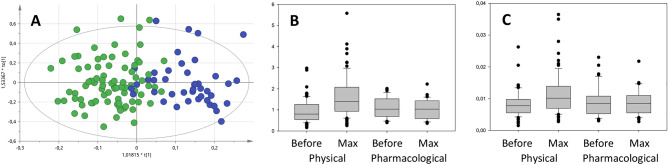
(**A**) corresponds to the OPLS-DA model differentiating exercise stress samples obtained before and at the time of maximum stress (Green dots: before; blue dots: maximum intensity). (**B**,**C**) correspond to the box plots of the concentrations of lactate and alanine, respectively.

Visual inspection of the spectra showed that exercise stress testing induced an increase in the lactate peak at around 1.35 ppm that returned to pre-test values after the test concluded, an increase that was not detected in the case of pharmacological stress. As shown in Fig. 3, a clear clustering in the exercise stress samples could be observed between pre-test and the time of maximum effort in OPLS-DA (Fig. 3A). However it was not possible to differentiate between pre-test and time of maximum effort in pharmacological stress. Furthermore, pharmacological samples obtained at the time of maximum effort tended to cluster with the pre-test exercise samples suggesting that no metabolic changes had happened during the test. Quantification of the metabolites confirmed that discrimination between pre-test and maximum effort relied on an increase in lactate and alanine (Fig. 3B,C) in exercise but not in pharmacological stress (Supplementary Table 3). There were no changes in the other metabolites quantified. These results confirm that the metabolic effects induced by exercise and pharmacological stress are markedly different, with potential consequences on the induction and interpretation of ischemia in patients undergoing pharmacological stress test.

### Prediction of stress test result

Among all patients undergoing pharmacological test, 20 had a positive result. Twenty-three matched controls were selected for comparisons. Similarly, 39 patients undergoing exercise stress test had a positive result, for which 44 matched controls were selected. Characteristics of patients in all groups are presented in Table 4. Cases and controls were similar with regards to demographics and past medical history, except for the presence of prior ischemic heart disease, which was higher in cases than in controls both in the exercise (*P* = 0.03) and the pharmacological stress test groups (*P* < 0.01).

**Table 4:** Serum metabolite concentration (mmol/L) according to the type of exercise; measured before and at the time of maximum intensity.

Metabolite	Exercise			Pharmacological		
	Before	Maximum intensity	*p*	Before	Maximum intensity	*p*
3-Hydroxybutyrate	0.044 ± 0.027	0.039 ± 0.025	0.591	0.043 ± 0.021	0.044 ± 0.023	0.994
Acetate	0.021 ± 0.014	0.022 ± 0.016	0.898	0.027 ± 0.013	0.094 ± 0.406	0.994
Alanine	0.008 ± 0.004	0.011 ± 0.006	0.0006	0.009 ± 0.005	0.009 ± 0.004	0.994
Betaine	0.021 ± 0.015	0.021 ± 0.015	0.898	0.024 ± 0.021	0.026 ± 0.020	0.994
Creatine	0.014 ± 0.010	0.014 ± 0.010	0.898	0.014 ± 0.008	0.016 ± 0.013	0.994
Creatinine	0.027 ± 0.016	0.027 ± 0.016	0.898	0.042 ± 0.037	0.042 ± 0.035	0.994
Glucose	1.725 ± 1.048	1.608 ± 1.028	0.898	1.980 ± 0.970	1.982 ± 0.980	0.994
Glycine	0.019 ± 0.029	0.013 ± 0.018	0.448	0.020 ± 0.021	0.020 ± 0.011	0.994
Isoleucine	0.063 ± 0.037	0.062 ± 0.037	0.898	0.069 ± 0.032	0.072 ± 0.041	0.994
Lactate	0.934 ± 0.548	1.599 ± 0.970	2.3 10^−6^	1.082 ± 0.488	1.025 ± 0.461	0.994
Threonine	0.135 ± 0.085	0.145 ± 0.088	0.898	0.163 ± 0.075	0.164 ± 0.096	0.994
Valine	0.103 ± 0.062	0.099 ± 0.067	0.898	0.105 ± 0.051	0.109 ± 0.060	0.994

*P* values were corrected for multiple comparisons.

**Figure 4:**
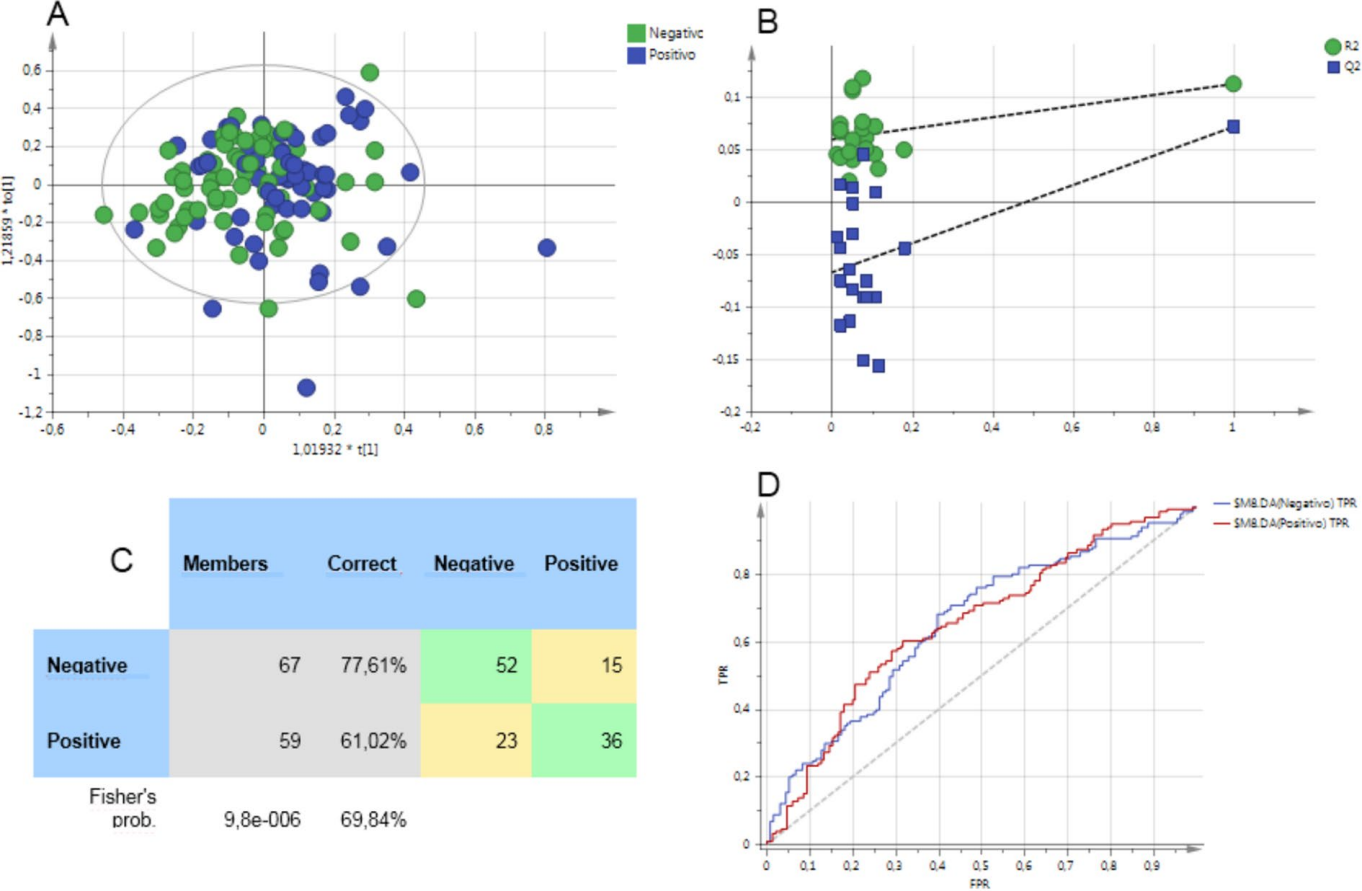
Pattern recognition results from the diffusion-edited spectra for the prediction of stress test results, (**A**) score plot corresponding to the OPLS-DA model able to predict the result of the stress test. (**B**) Corresponds to the permutation analysis, (**C**) is the misclassification table showing Fisher´s test and (**D**) the ROC Curve of the model shown in (**A**).

Using the samples obtained before the stress test, we created several models in order to evaluate the role of the metabolic profile in the prediction of the stress test results (positive or negative for ischemia). Models made with spectra that contained lipids were statistically significant according to Fisher’s exact test but only the one made with diffusion spectra had a *P* < 0.05 according to CV-ANOVA, and a classification success of 70% (Fig. 4).

The metabolic profile changed during stress, mainly in patients undergoing exercise stress test (see earlier), we evaluated the predictive ability of the metabolic profile by separating the two modalities of stress test. Interestingly it was possible to predict the result of stress test for pharmacological and exercise tests (P CV-ANOVA 0.014 and 0.033 respectively).

**Figure 5:**
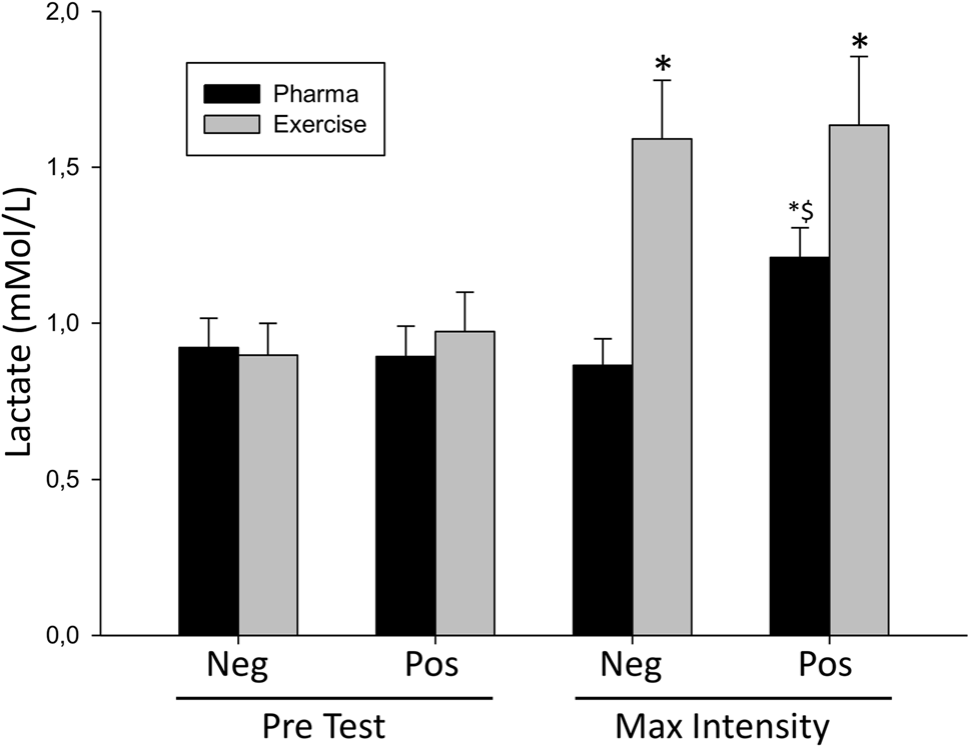
Serum lactate levels (mmol/L) in samples obtained before the test and at the time of maximum intensity. Comparison between positive and negative stress test results. *Indicates statistical difference (*P* < 0.05) from pre-test and ^$^ between negative and positive results.

Finally, because exercise stress induced an increase in lactate concentrations we evaluated if lactate behaved differently between positive and negative stress. Figure 5 shows serum lactate levels and their evolution during stress test for pharmacological and exercise patients. As shown above there is a large increase in lactate levels associated to exercise. It is also worth noting a clear increase in lactate associated positive but not negative cases of pharmacological testing.

The increase in lactate seen in pharmacological positive patients (25%) is lower than the increase associate to exercise (40%); thus it is likely that myocardial lactate is not detected in the global population of exercise patients as it may be diluted by the much larger effect of exercise. Also, in preliminary experiments using a large animal model (swine) we have seen an increase in peripheral blood lactate associated to myocardial infarction (Supplementary Fig. 2) demonstrating that the metabolic effects of myocardial ischemia are detectable in the bloodstream.

## Discussion

In the present work we have characterized a population that included patients with suspected CAD undergoing myocardial perfusion SPECT with physical or pharmacological stress test. The clinical and epidemiological features of the population under study are not different from other studies done in a similar environment^13,14,16,17^.

Of the confounding variables studied only sex and statin treatment did have an effect on the serum metabolic profile. Sex does affect the shape of the lipid peaks in the spectrum which is consistent with men and women having different lipoprotein profiles^11^. It has been described that women have smaller-sized VLDL and larger sized LDL and HDL than men leading to a less atherogenic profile^18^. However, sex differences are not limited to lipoproteins as they extend to some of the small metabolites present in serum as we have described in a preliminary report^19^. Among the low molecular weight metabolites, glycine concentration was found to be higher in women than men. Glycine has been shown to be protective against ischemia–reperfusion injury^20^, and has been suggested to be involved in the protective effects of remote ischemic conditioning^21^. These findings might explain, at least in part, the fact that women have smaller infarct size once corrected for confounding factors^22^.

Statins are drugs that reduce the cholesterol levels and are able to reduce cardiovascular risk^23^. The effect of statin treatment on the metabolic profile could be detected only in diffusion-edited and pulse-and-acquire spectra that contain the full lipoprotein envelope. The main differences were found at 1.28 and at around 2.0 ppm, the area were lipid double bonds appear. Changes in the 1.28 ppm had previously been reported^11^ and our findings regarding the double bonds are in accordance with previous findings that statins change lipid composition but not circulating amino acids, ketones, or glycolysis-related metabolites^24^.

Other risk factors evaluated in population screening such as diabetes dyslipidemia or age did not affect the metabolic profile in the cohort under study. Numerous metabolomics studies involving diabetic patients^25,26^ and diabetic cardiomyopathy^27^ have shown differences associated to the disease. In our study, one third of the patients included were diagnosed as diabetics; however, they were treated with insulin or oral antidiabetic agents and none had glucose levels above 6 mmol/L at the time of analysis suggesting that the disease was under control which could explain the lack of metabolic effect associated to diabetes.

Likewise, none of the spectra showed a clear dyslipidemic pattern although 84.8% of patients had been previously diagnosed of dyslipidemia, a similar percentage to other studies^13,15,28^. Most of the patients diagnosed as dyslipidemics in our study were treated with statins as the drugs of first choice recommended for patients with hypercholesterolemia or combined hyperlipidemia^29^.

Age has also been described as a factor affecting the metabolic profile of blood^30,31^ but it was not a relevant factor modifying the metabolic pattern in our study. Furthermore, although patients performing pharmacological stress test were older than exercise stress patients, it was not possible to obtain a discriminant model to differentiate them nor could we detect age based differences. These apparent discrepancies may be explained by the lack of young patients in our study population.

Physical stress induced the accumulation of lactate and alanine in the serum, in agreement with previous studies done with mass spectrometry^13^. On the other hand, pharmacological testing did not induce changes in the metabolic profile. Furthermore, samples taken at the time of maximum intensity of pharmacological stress test group tended to cluster with the samples taken before exercise stress test. This is in accordance with pharmacological stress test actually detecting myocardial unbalanced blood redistribution in the presence of stenosis^32^. In view of those results, we urge caution in the interpretation of pharmacological stress testing results as the use of vasodilators does not fully mimic the regional ischemic effect seen in exercise testing.

The value of NMR spectroscopy-based metabolic profiling to predict the occurrence of exercise-induced myocardial ischemia in patients with CAD was previously evaluated by our research group^14^ using a permutation approach. Although in the present study, we have been able to obtain models to predict the occurrence of ischemia in our population and similarly to previously published results^14^ these models are statistically significant when evaluated using Q^2^ or Fisher’s probability test that has been proposed as a better model for biomarker discovery^33^. However, only diffusion edited spectra was able to reach statistical significance when the models where evaluated with CV-ANOVA, a more restrictive statistical approach^34^. In view of these results, a new study with increased number of patients (and power) should be performed.

It is interesting to note that in the case of pharmacological testing we could detect an increase in lactate associated to positive stress results. We hypothesize that this lactate originates in the heart; an idea supported by the fact that in a large animal model we are able to detect an increase in lactate in peripheral blood associated to myocardial infarction. In the case of physical stress testing, myocardial lactate increase would be masked due to the fact that exercise does induce a large increase in lactate most likely of muscular origin. The presence of myocardial lactate would suggest that pharmacological testing does induce ischemia, not only blood supply redistribution.

The results presented in this work showed a 70% sensitivity in the detection of myocardial ischemia using a metabolomic approach. These results are similar to previous findings that tried to predict the occurrence of death after myocardial infarction^35^. However, metabolic profile provided added value on top of other classical risk stratification approaches such as the GRACE score.

Finally, we are aware that stress testing and clinical evaluation of the patients will be the technique of choice for the prediction of ischemia in the foreseeable future. However, any approach that could reduce the burden placed on health systems would be very welcome in this role if, for example, a strategy with high negative predicting value could be devised or if it could be applied with high sensitivity to a defined population such as younger patients with reduced comorbidities.

In conclusion, we have been able to characterize the metabolic profile of patients at risk of cardiovascular events. We have established that pharmacological stress test does not reproduce the dynamic changes observed in exercise stress but it is very likely able to induce myocardial ischemia; however, care should be taken when evaluating clinical results. Finally, although there is promising evidence suggesting that ^1^H NMR based metabolomics could predict stress test results this study was underpowered to obtain a definitive answer.

## Experimental

### Patients and study design

The study was prospectively conducted in 244 consecutive patients with suspected cardiovascular risk who were referred at Hospital Vall d`Hebron to perform a myocardial perfusion SPECT study either with exercise stress testing on a treadmill (Bruce protocol), or with pharmacological stress with dipyridamole or regadenoson. Patients had to be older than 18 year old, and were included in the Metabolomic Profile of Patients Undergoing Myocardial Perfusion SPECT (METS- Clinicaltrial.gov identifier: NTC0298771) study. Patients unable to sign the written consent, pregnant women and patients with dobutamine indication in the stress protocol were excluded.

The study embraced four groups of patients. Patients were divided into two groups depending on the type of stress test to which they were subjected (exercise or pharmacological). Within each group, two subgroups were defined according to the result reported by the SPECT: cases were patients whose result was positive for myocardial ischemia and controls were those with negative result. All cases were included in the study, whereas age and sex-matched controls were selected for both exercise and pharmacological groups.

The study was approved by the Hospital Vall d´Hebron´s ethics committee, all patients gave their written informed consent to be included and all methods were performed in accordance with the relevant guidelines. Patients were interviewed, their clinical records examined, and information on their medical history retrieved from medical records.

### Samples

Fasted venous blood samples (5 ml) were obtained before stress test, at the time of maximum intensity and after the test for each patient using the same intravenous line used for radiopharmaceutical and coronary vasodilator in the case of pharmacological stress test. Blood was allowed to clot for two hours at room temperature, then, the serum was collected by centrifugation at 1000*g*, 4 °C, for 5 min and stored at − 80 °C until assayed^19^.

### Serum deproteinization

Serum deproteinization was done using methanol precipitation according to protocols described previously^36^. Briefly, 200 µl of serum were mixed with 400 µl of methanol, vortexed, and kept at − 20 °C for 20 min. Afterwards, the mixtures were centrifuged at 11,000*g*, 4 °C, for 30 min. The supernatants were recovered, dried under a stream of nitrogen and stored at − 80 °C until NMR analysis.

### NMR spectroscopy

For whole serum samples, 200 µl of serum were diluted with 300 µl of phosphate-buffered saline (PBS) at pH 7.4 prepared with D_2_O and placed in 5 mm NMR tubes for analysis. A series of spectra including: (1) pulse-acquire sequence preceded with WATERGATE 3-9-19, (2) a CMPG with an effective T2 delay of 32 ms, and (3) diffusion edited spectra were acquired for each sample^19^. CPMG and diffusion edited sequences were preceded by presaturation of the water signal for 2 s. Each spectrum consisted in the accumulation of 64 scans and lasted approximately 5 min.

In the case of deproteinized serum, dried samples were reconstituted in 500 µl of PBS made up with D_2_O, containing 0.5 mM trimethylsilyl tetradeuteropropionic acid sodium salt (TSP) as a concentration and chemical shift reference and placed in a 5 mm NMR tube^19^. Each spectrum consisted in the accumulation of 64 scans with NOESYPR1D pulse sequence with a mixing time of 100 ms. Spectra were obtained at 300 K on a 400 MHz vertical bore magnet interfaced to a Bruker Avance console.

### Pattern recognition

Prior to pattern recognition each spectrum was manually phase corrected, normalized to a total area of 1 and variables pareto scaled. The area between 0.5 and 9 ppm (excluding the water zone) were divided into bins of equal width of 0.01 ppm and imported in SIMCA (Umetrics, Umea, Sweeden) software version 14.0 for further analysis. A non-supervised classification approach such as Principal Component Analysis (PCA) was used. When PCA was not able to cluster the samples, Orthogonal Projection to Latent Structures Discriminant Analysis (OPLS-DA), a supervised analysis, approach was used. Discriminant models were considered statistically significant when CV-ANOVA was < 0.05.

Metabolite quantification was performed in spectra of desproteinized samples using Chenomx software (Chenomx, Inc. Edmonton, AB, Canada) by comparing de areas of the peaks of interest to that of TSP added as an internal standard at a final concentration of 0.5 mM.

### Statistical analysis

Demographic and clinical data processing was carried out using statistical package SPSS version 21.0. Categorical variables were summarized by absolute and relative frequencies. The Kolmogorov–Smirnov test confirmed the normal distribution of all continuous variables collected in the study, which were reported as mean ± standard deviation (SD). Categorical variables were compared with the X^2^ test. Continuous variables were compared with the Student *t* test. *P* value was considered statistically significant when was < 0.05. *P* values were corrected for multiple comparisons when appropriate.

## Supplementary information

Supplementary Information.
