# Supplementary material for: 1H NMR serum metabolomic profiling of patients at risk of cardiovascular diseases performing stress test
Source: Sci Rep. 2020 Oct 20;10:17838. doi: 10.1038/s41598-020-74880-6 (PMC7575600; doi:10.1038/s41598-020-74880-6)

^Supplementary Material^

**^1^H NMR Serum metabolomic profiling of patients at risk of cardiovascular diseases performing stress test.**

Camila Lema^a^, Mireia Andrés^a^, Santiago Aguadé^a^, Marta Consegal^a,b^, Antonio Rodriguez-Sinovas^a,b^, Begoña Benito^a,b^, Ignacio Ferreira-Gonzalez^a,c^, Ignasi Barba^a,b,d^

^a^Cardiovascular Diseases Research Group, Department of Cardiology, Vall d’Hebron University Hospital and Research Institute, Universitat Autònoma de Barcelona, Barcelona, Spain.

^b^ Centro de Investigación Biomédica en Red sobre Enfermedades Cardiovasculares (CIBER-CV), Spain.​

^c^ Centro de Investigación Biomédica en Red sobre Epidemiología y Salud Pública (CIBERESP), Spain

^d^ Vall d’Hebron Insitutute of Oncology (VHIO), Barcelona, Spain

**Supplementary Table 1**. Epidemiological and clinical characteristics of patients according to cases and controls for each stress test groups.

| **Characteristics** | **Physical stress** | | |  | **Pharmacological stress** | |  |
| --- | --- | --- | --- | --- | --- | --- | --- |
|  | N=83 | | | **p** | N=43 | | **p** |
|  | Cases | | Controls |  | Cases | Controls |  |
|  | (N=39) | | (N=44) |  | (N=20) | (N=23) |  |
| **Demographics** |  | |  |  |  |  |  |
| Men | 35 (89.7%) | | 38 (86.4%) | 0.74 | 11 (55.0%) | 12 (52.2%) | 0.85 |
| Age (years)* | 63.5 ±11.4 | | 64.1 ± 9.0 | 0.77 | 73.0 ±7.9 | 74.2 ±9.6 | 0.64 |
| **Cardiac risk factors** | | | | | | | |
| Smoking | (n=30) | | (n=44) |  | (n=15) | (n=23) |  |
| Ex smoker | 19 (63.3%) | | 27 (62.8%) |  | 8 (53.3%) | 8 (36.4%) |  |
| Smoker | 4 (13.3%) | | 4 (9.3%) | 0.82 | 3 (20.0%) | 1 (4.5%) | 0.10 |
| Non smoker | 7 (23.4%) | | 12 (27.9%) |  | 4 (26.7%) | 13 (59.1%) |  |
| Hypertension | 25 (64.1%) | | 27 (61.4%) | 0.48 | 17 (85.0%) | 16 (69.6%) | 0.57 |
| Dyslipidemia | 34 (87.2%) | | 38 (86.4%) | 0.87 | 18 (90.0%) | 18 (78.3%) | 0.10 |
| Diabetes | 13 (33.3%) | | 10 (22.7%) |  | 11 (55.0%) | 8 (34.8%) |  |
| Non insulin requirement | 10 (76.9%) | | 6 (60.0%) | 0.38 | 4 (36.4%) | 6 (75.0%) | 0.10 |
| Insulin requirement | 3 (23.1%) | | 4 (40.0%) |  | 7 (63.6%) | 2 (25.0%) |  |
| **Prior cardiovascular disease** | | |  |  |  |  |  |
| Ischemic heart disease | 26 (66.7%) | | 19 (43.2%) | 0.03 | 13 (65.0%) | 5 (21.7%) | <0.01 |
| Coronary revascularization | 25 (96.2%) | | 15 (34.1%) | 0.02 | 9 (69.2%) | 3 (60.0%) | 0.05 |
| **Long-term medications** | | | | | | | |
| Statins | 33 (86.8%) | | 32 (72.7%) | 0.12 | 16 (80.0%) | 15 (65.2%) | 0.28 |
| ASA | 31 (79.5%) | | 33 (75.0%) | 0.80 | 11 (55.0%) | 13 (52.2%) | 0.92 |
| Beta-blockers | 25 (64.1%) | | 21 (47.7%) | 0.11 | 14 (70.0%) | 12 (52.2%) | 0.23 |
| Nitrates | 11 (28.2%) | | 5 (11.4%) | 0.09 | 8 (40.0%) | 3 (13.0%) | 0.07 |
| Oral antidiabetics | 34 (84.6%) | | 10 (20.5%) | 0.29 | 8 (35.0%) | 7 (26.1%) | 0.54 |
| **Antropometric and Laboratory parameters*** | | | |  |  |  |  |
| IBM | 28.5± 3.8 | 27.94 ± 3.9 | | 0.56 | 31.2 ± 5.7 | 29.6 ± 5.6 | 0.37 |
| Cholesterol (mg/dl) | 160.8± 36.1 | 182.7± 42.0 | | 0.01 | 172.7± 53.3 | 188.2 ±41.0 | 0.29 |
| LDL (mg/dl) | 91.4± 31.2 | 110.3± 38.7 | | 0.02 | 93.8± 38.9 | 105.6 ±32.3 | 0.29 |
| HDL (mg/dl) | 45.7±10.1 | 47.3± 12.9 | | 0.55 | 45.4± 15.0 | 52.5 ±11.3 | 0.09 |
| Triglycerides (mg/dl) | 119.8± 41.5 | 122.2± 43.0 | | 0.80 | 171.7± 74.3 | 131.6 ±55.7 | 0.05 |
| Glomerular filtration (mL/min/1.73 m2) | 81.8 ± 13.4 | 78.6± 16.5 | | 0.35 | 58.3± 27.0 | 67.1± 22.5 | 0.27 |
| **SPECT data** |  |  | |  |  |  |  |
| SPECT indication |  |  | |  |  |  |  |
| Diagnostic | 11 (28.2%) | 26 (59.1%) | | 0.01 | 4 (20.0%) | 16 (69.6%) | <0.01 |
| Pronostic | 28 (71.8%) | 18 (40.9%) | |  | 16 (80.0%) | 7 (30.4%) |  |
| METS* | 7.9 ±1.9 | 8.3 ±2.1 | | 0.40 | - | - | - |
| Initial BP (mmHg)* | 135.9 ±20.5 | 134.4 ±17.1 | | 0.71 | 142.1 ± 24.0 | 145.3 ± 25.8 | 0.67 |
| Maximum intensity BP (mmHg)* | 166.5 ±33.5 | 168.4 ±30.9 | | 0.79 | 135.8 ± 22.1 | 142.1 ±21.3 | 0.34 |
| Basal HR (rpm)* | 70.4 ±16.5 | 72.0 ±15.2 | | 0.66 | 72.3 ± 15.8 | 70.0 ± 13.5 | 0.62 |
| Maximal HR (rpm)* | 125.3 ±20.0 | 130.0 ±21.0 | | 0.30 | 91.2 ± 19.8 | 88.7 ± 15.5 | 0.65 |
| % Increase HR* | 84.6 ±44.1 | 86.2 ±39.7 | | 0.86 | 27.6 ± 21.6 | 28.6 ± 19.2 | 0.88 |
| %FE* | 51.8 ±11.3 | 58.0±10.3 | | 0.01 | 52.8 ± 13.5 | 61.5 ± 11.0 | 0.03 |
| *Mean (SD) |  |  | |  |  |  |  |

**Suppmementary Table 2.** Blood serum metabolite concentration according to statin use

| **Metabolite** | **Stantins** | **No statins** | **p** |
| --- | --- | --- | --- |
| 3-Hydroxybutyrate | 0.042 ± 0.024 | 0.049 ± 0.028 | 0.216 |
| Acetate | 0.023 ± 0.014 | 0.022 ± 0.011 | 0.763 |
| Alanine | 0.008 ± 0.004 | 0.009 ± 0.005 | 0.134 |
| Betaine | 0.022 ± 0.019 | 0.022 ± 0.012 | 0.946 |
| Creatine | 0.013 ± 0.008 | 0.016 ± 0.013 | 0.130 |
| Creatinine | 0.034 ± 0.028 | 0.027 ± 0.014 | 0.871 |
| Glucose | 1.792 ± 0.913 | 1.857 ± 1.321 | 0.760 |
| Glycine | 0.017 ± 0.021 | 0.026 ± 0.034 | 0.075 |
| Isoleucine | 0.064 ± 0.036 | 0.069 ± 0.034 | 0.542 |
| Lactate | 0.965 ± 0.502 | 1.056 ± 0.625 | 0.420 |
| Threonine | 0.144 ± 0.081 | 0.147 ± 0.081 | 0.869 |
| Valine | 0.102 ± 0.059 | 0.112 ± 0.056 | 0.420 |

**Tables showing the characteristics of the various OPLS-DA models obtained in this work.**

Models obtained to differentiate between patients that used statins from those that did not use them

| Sequence | R2X | R2Y | Q2 | Fisher | CV ANOVA |
| --- | --- | --- | --- | --- | --- |
| WG | 0.36 | 0.12 | 0.10 | 0.026 | 0.002 |
| CPMG | 0.21 | 0.05 | -0.004 | 0.41 | 1 |
| Diffusion | 0.847 | 0.368 | 0.18 | 7.4^-10^ | 0.003 |
| NOESYPR1D | 0.47 | 0.04 | 0.001 | 0.23 | 0.92 |
|  |  |  |  |  |  |

Models obtained to differentiate between samples taken before and samples taken at the maximum intensity of physical stress test

| Sequence | R^2^X | R^2^Y | Q^2^ | Fisher | CV ANOVA |
| --- | --- | --- | --- | --- | --- |
| WG | 0.72 | 0.56 | 0.45 | 1.8^-28^ | 8.85^-17^ |
| CPMG | 0.43 | 0.56 | 0.49 | 3.20^-28^ | 8.05^-21^ |
| Diffusion | No fit | No fit | No fit | No fit | No fit |
| NOESYPR1D | 0.67 | 0.32 | 0.23 | 5.2^-18^ | 1.42^-8^ |
|  |  |  |  |  |  |

Models obtained to differentiate between samples taken before and samples taken at the maximum intensity of pharmacological stress test

| Sequence | R^2^X | R^2^Y | Q^2^ | Fisher | CV ANOVA |
| --- | --- | --- | --- | --- | --- |
| WG | 0.59 | 0.532 | 0.20 | 2.3^-7^ | 0.01 |
| CPMG | 0.48 | 0.28 | 0.10 | 1.90^-6^ | 0.07 |
| Diffusion | No fit | No fit | No fit | No fit | No fit |
| NOESYPR1D | No fit | No fit | No fit | No fit | No fit |
|  |  |  |  |  |  |

Models obtained to differentiate between physical and pharmacological stress test groups at the time of maximum intensity.

| Sequence | R^2^X | R^2^Y | Q^2^ | Fisher | CV ANOVA |
| --- | --- | --- | --- | --- | --- |
| WG | 0.74 | 0.69 | 0.53 | 7.5-^25^ | 6.05^-15^ |
| CPMG | 0.46 | 0.63 | 0.55 | 4.30-^18^ | 1.68^-18^ |
| Diffusion | 0.76 | 0.24 | 0.15 | 1.1^-6^ | 0.004 |
| NOESYPR1D | 0.61 | 0.37 | 0.24 | 1.3^-8^ | 8.99^-7^ |
|  |  |  |  |  |  |

**Supplementary Table 3.** Metabolite concentration (mmol/L) derived from deproteinized spectra samples taken before and at the moment of maximum intensity in physical stress test group.

| **Metabolite** | **Physical Stress Test** | | | **Pharmacological Stress Test** | | |
| --- | --- | --- | --- | --- | --- | --- |
|  | **Pre-Test** | **Max Intensity** | **p** | **Pre-Test** | **Max Intensity** | **p** |
| 3-Hydroxybutyrate | 0.044 ± 0.027 | 0.039 ±0.025 | 0.197 | 0.043 ± 0.021 | 0.044 ± 0.023 | 0.840 |
| Acetate | 0.021 ±0.014 | 0.022 ±0.016 | 0.715 | 0.027 ± 0.013 | 0.094 ± 0.406 | 0.281 |
| Alanine | 0.008±0.004 | 0.011 ±0.006 | <0.01 | 0.009 ± 0.005 | 0.009 ± 0.004 | 0.933 |
| Betaine | 0.021±0.015 | 0.021 ±0.015 | 0.789 | 0.024 ± 0.021 | 0.026 ± 0.020 | 0.730 |
| Creatine | 0.014±0.010 | 0.014 ±0.010 | 0.889 | 0.014 ± 0.008 | 0.016 ± 0.013 | 0.389 |
| Creatinine | 0.027±0.016 | 0.027 ±0.016 | 0.898 | 0.042 ± 0.037 | 0.042 ± 0.035 | 0.933 |
| Glucose | 1.725±1.048 | 1.608 ±1.028 | 0.469 | 1.980 ± 0.970 | 1.982 ± 0.980 | 0.994 |
| Glycine | 0.019±0.029 | 0.013 ±0.018 | 0.112 | 0.020 ± 0.021 | 0.020 ± 0.011 | 0.948 |
| Isoleucine | 0.063±0.037 | 0.062 ±0.037 | 0.87 | 0.069 ± 0.032 | 0.072 ± 0.041 | 0.718 |
| Lactate | 0.934±0.548 | 1.599 ±0.970 | 1.943 ^-7^ | 1.082 ± 0.488 | 1.025 ± 0.461 | 0.579 |
| Threonine | 0.135±0.085 | 0.145 ±0.088 | 0.453 | 0.163 ± 0.075 | 0.164 ± 0.096 | 0.969 |
| Valine | 0.103±0.062 | 0.099 ±0.067 | 0.75 | 0.105 ± 0.051 | 0.109 ± 0.060 | 0.730 |

**Supplementary Figure 1.**

**A**

**B**

1. OPLS-DA score plot for the discrimination of patients taking statins (green dots) from those who did not (blue dots) obtained using pulse and acquire spectra. B) corresponds to the s-plot around the methyl lipid peaks marked with red circles are the most relevant variables in the discrimination; note that those variables are at the top of the peak.

**Supplementary Figure 2.**

Lactate concentration measured form the coronary synus (black dots) peripheral blood (white dots) using NMR spectroscopy in swine undergoing myocardial infarction by ligation of the anterior descendant artery. Samples were obtained prior to artery ligation (basal) and 1, 5, 10 and 120 minutes after the opening of the artery.

**Supplementary Figure 3.**

Paired analysis of the effects of stress testing showed that lactate (peak at 4.11 ppm) behaved differently between men and women.


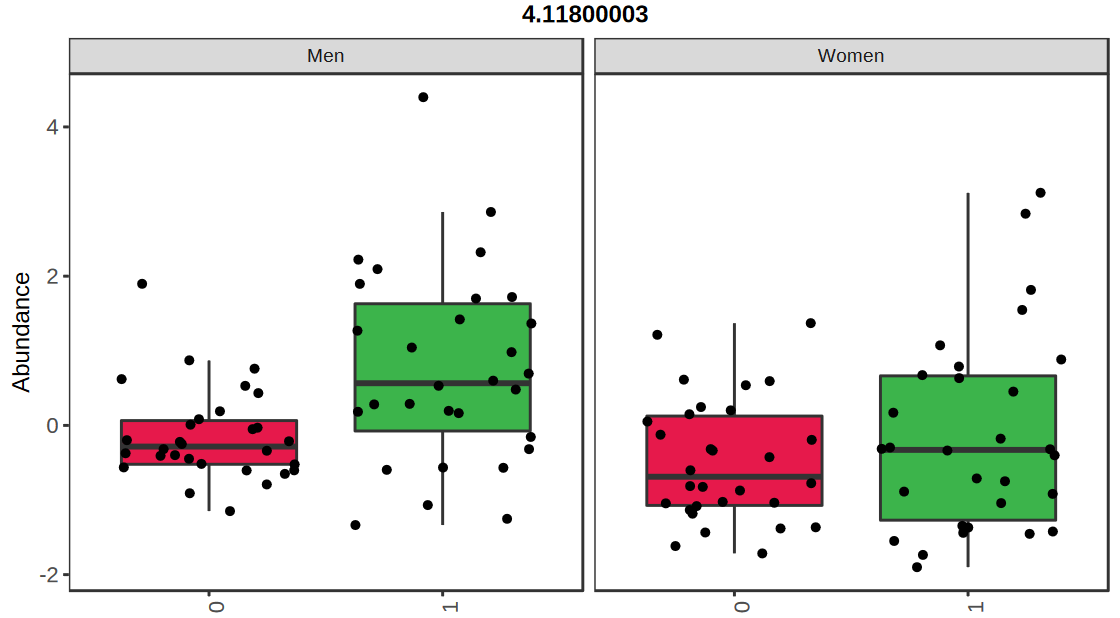

Supplement: Supplementary file 1 — Supplementary Information. [file 41598_2020_74880_MOESM1_ESM.docx]
